# The General and the Special in Medical Practice

**Published:** 1955-11

**Authors:** E. M. Bluestone

**Affiliations:** Consultant, Montefiore Hospital, New York City


### THE GENERAL AND THE SPECIAL IN MEDICAL PRACTICE

BY

E. M. BLUESTONE, M.D.

*Consultant, Montefiore Hospital, New York City*

Differentiation and the division of labour, which characterized the Industrial Revolution and influenced the progress of civilization, have been at work in the practice of medicine. Whatever value one may place on the logic of these phenomena, the effect cannot be the same in medicine where there is no such thing as independent tissue, and in industry where there is. In the one case we relate man to man and in the other we relate man to a machine or part of a machine. The mechanistic conception of medical practice may have had its day when such specialities as modern surgery and physical medicine were first hailed for their technical excellence. This conception has been modified however under the influence of the psychosomatic principle which forbids the segmentation of any sick person in the presence of his physicians. We have come full circle now with the realistic application of the fundamental truth governing the relation of body and mind to each other. The most recent of the medical sciences, namely, Social Medicine, is in fact nothing more than the aggregate of these new concepts on behalf of the individual in relation to his environment. We can and should differentiate, and see that labour is divided efficiently among specialists, but they must be within sight and hearing of each other if the result is to be constructively synthetic. Anything that willl neutralize the handicap of illness in the struggle for existence and reduce "man's inhumanity to man" should be welcome.

Though all-pervasive, the practice of medicine has not yet reached its full organizational growth and social potentialities. There is something wrong with the interpretation of "general" and "special", to give only one example. How can we successfully select and apply the appropriate techniques of industry and its factories, of commerce and its market place, to medical practice? How can we modify them, and provide them with any safeguards that might be necessary to protect all parties to the arrangement? Specifically, what is the meaning and the practical significance of the general and the special in the private and public practice of medicine, and of the general hospital and the special hospital? Can we draw up rigid terms of reference for each of them? Is there a sharp line of demarcation and, if there is, where approximately is it located and how is it identified, assuming that its point in space can be fixed with a reasonable degree of accuracy? If there is not, how can we achieve flexibility so that the general and the special may be blended in accordance with the requirements?

As we continue in this questioning mood we find ourselves invading the territory assigned to, or assumed by, the general practitioner of medicine on the one hand, and the specialist (sometimes referred to as consultant, when he is not in direct control of the patient) on the other; of the general hospital and of the special hospital and the traditions which restrain them. The psychology of the "empire-builder" must be taken into consideration. Have we been drifting in response to immediate social and medical pressures and tensions, meeting situations as they arise and responding in direct proportion to the urgency of the call, or have we been planning with more permanent gains in view, in a balanced effort to achieve stability without sacrificing flexibility? Urgency of the call, I might add, though attractive as a stimulant to planning, is inadequate because it is too often temporary, subjective, unreliable, subdued, and dependent on the strength (or feebleness) of the caller. Planning must go far beyond the acute stage of illness.

What, specifically, is the domain of general practice and what of the specialities or consulting services ? I remind my readers here that we are labouring in a field of thought where the principles of medical care are eternal and therefore universally applicable. Translated into practice they should be independent of medical and hospital economics, in the sense that what is best for the patient, within reason, is best and least expensive in the long run for the community in which he lives and to which he contributes when he is well enough to do so. Whether the patient pays his own way, shares the cost of medical care with philanthropy, or is covered by a national policy of medical care, his needs remain the same. In Great Britain, the United States, or Russia, three countries in which methods differ radically from each other, the identity and interdependence of general and special must remain the same. We are therefore free to state principles based on a staggered list of requirements, depending not so much upon the subjective urgency of the call, as upon the need as it is seen objectively. When a man modifies the strength of his call in response to fear of the fee-for-service principle he may be imposing a later burden on his community. This becomes a penalty for the inaction of the moment. The success of an organization may be seen in its communal usefulness and the lessons which it teaches may be learned in a positive as well as in a negative way. It surprises me that hospitals, general and special, do not devote as much time and energy to the analysis and interpretation of the work which they do not perform—which they actually reject—as they do to the work which they welcome and perform. It is quite as important to know in what respects the community in which the hospital functions (including its doctors) is served, as it is to know in what respects it is not served—and why.

The general practitioner, in all cases where the situation is justifiably under his control, is presumed to be in possession of the fundamentals of medical knowledge for everyday use, supplemented by sufficient information about the specialties to enable him to decide when he can proceed under his own power, when to share responsibility, and when to transfer it. He is closest to the patient over a longer period of time and the evidence which he gives should therefore be respected. It is literally true that if the practitioner were not in existence he would have to be created. He is indispensable, and not expendable. As an inheritor of the traditions of healing he has borne the torch of medical learning through the ages. Since his group does not, as a rule, belong to the creative echelons of scientific medical effort, there is all the more need for bringing together teacher and student in this useful area of human activity. The general practitioner derives his skills from a combination of teaching specialists. If the practitioner has not adjusted, or been adjusted, to the time, place and other circumstances in which he must pass his professional life, who shall say that the fault is his alone? Has opportunity for continuous interplay between general and special, and between routine and research, been solidly based on sound organization and administration, and has it been offered to him under a more promising system of rewards and punishments? The arrangements must be such as to enable us to exploit the services of the practitioner to the limit. Can we report that scientific opportunities have corresponded in reasonable measure to the available talent in general practice? Have not our hospitals closed their eyes and ears, as well as their doors, to his voiced—and unvoiced—needs, often condemning him to practice medicine based almost entirely on memories going back to medical-school days? What can it profit us if we qualify him and send him forth forever endowed with the legal right to depend almost entirely on the knowledge which he has acquired up to the date of the issuance of his diploma. Educational responsibility cannot end here. In spite of standardizing agencies, certification for special qualifications, and other controls, he is still under the protection of the law, the while he is beset by inducements, temptations and distractions which too often lead to petty if not gross malpractice.

My emphasis here is on the hospital, and the highly placed staff which gives it standing, in relation to the practitioner who is compelled to carry on alone under the smarting disregard which is so frequently his portion. Another point for memory here. An insecure physician means an insecure patient, and insecurity refers not alone to financial income but also to educational adequacy. It is wrong to make invidious comparisons between general practitioner and consultant-specialist based on relative position with respect to the hospital.

Those who feel that the wider distribution of the educational opportunities of the hospital to put an end to the isolation and current inadequacies of the suppressed practitioner would be "caviare to the general" have prejudged the case and come to a hasty conclusion. Such a verdict of condemnation would have to be supported by substitute ideas of a constructive nature. What would the plan of organization then be and how would it be maintained? The frustrations of the practitioner are only partly of his making and are based on the difficulty of maintaining a bold front in the presence of the patient while a hostile, though well placed, minority in the medical profession looks on with uplifted eyebrows. Yet it is an elementary rule of pedagogy that resistance in the student caused by ridicule, sarcasm, or failure to understand the relationship can only be overcome in one obvious way. *There is more than a hint of the remedy in the thought that every bed in every hospital, no matter where it is located or by whom occupied, is potentially a teaching bed and potentially a research bed.*

We are all familiar with the pathologist who has been endowed by the creator with hindsight which enables him in unguarded moments to hint somewhat carelessly about the inferiority of those who missed their opportunities during the lifetime of the patient. His special diagnostic skill is admittedly superior to that of the physician whose ability to penetrate medical mysteries may be limited by the facts of life. Boastfulness comes naturally to the favoured worker who is successful, but it can be distressingly irritating when it is exhibited in the presence of a colleague who, because of his natural limitations, has appealed to him for help. Between the post-mortem diagnostician who is uninhibited in his search for the truth and not exposed to the pitfalls of prophecy on the one hand, and the consultant-specialist who has the advantages of freshness, basic preparation of the case, and the prized opportunity of maintaining his skills and prestige as well as intensive training in a more favourable environment on the other, the practitioner is fortunate who can survive with dignity and self-respect. What I am trying to say here is that he must be judged more understandingly, more sympathetically and, above all, more helpfully, by those who are better placed in medical life and who must be called in when he has come to the end of his line.

What do we find on the other side of the picture as we analyse the absolute and relative position of the consultant-specialist? Assuming that the outline of his duties has been accepted, which is not often the case—witness the current dispute between the position of the internist and the paediatrician with respect to the stage of childhood, or the status of nose-and-throat medicine in an antibiotic age—what allowance is made for overlapping of activity and how much for constriction of view? The general practitioner who eventually narrows his professional activity down to one specialty should not thereby move into the rarefied atmosphere of the ivory tower. It is significant that the reverse movement, from special to general practice, almost never takes place. Once a specialist always a specialist, and here too the lesson is that we must not only have respect for selective interest on the part of qualified men, but for general interest on the part of those who are gifted that way. Whatever cleavage may prevail socially between the less remunerated and the more remunerated must not be carried over into the area of medical activity for we should then be placing a false premium on the services of the man whose fee is higher, and particularly in the presence of a patient who judges therapeutic values in this quaint way. We shall not understand the relationship fully if we condone and perpetuate a second-class status for the practitioner. The man who plunges directly into a speciality without general preparation is even more handicapped, unless he is temperamentally adjusted to the presence of the general practitioner on strategic occasions.

We have seen the rise of specialization as each of the sciences was applied to the practice of medicine. The physician who discovered early in life, and sometimes later, that he had mechanical talent, that he had nimble fingers which could successfully put damaged parts together, applied his energies to the field of surgery, general or special. The physician who discovered that he was good at chemistry, headed for the green pastures of internal medicine and some of its sub-specialities in particular. The one who had a flair for physics found himself in the area covered by physical medicine.

The mathematician among us took a long look at biostatistics and sometimes stayed with it. The one who saw possibilities in botany and zoology gravitated toward bacteriology and sometimes pathology. In each case where talent was rewarded knowledge flourished happily—sometimes oblivious of the fact that general practice is based on gifts of its own which may be of wider range, if not specifically definable, in the presence of a non-specific clientele. The untutored activity of the bone-setter is frowned upon by educated specialists—and for the excellent reason that the one is educated to his task while the other acts by instinct—yet both share a common demominator of selective interest. There is also the man who narrows his special proclivities down to the ludicrous pin-point of human anatomy or behaviour. A prominent wag who flourished in the last generation and disapproved of the ever-widening tendency toward specialization, spoke of "specialists of the linea alba" which he foresaw in his humorous way. My late teacher, Cornelius Coakley, once told us, with a twinkle in his eye, that he had examined 100,000 nasal septa in his lifetime and had never found one that was straight! He was talking to future general practitioners, with a sprinkling of future nose-and-throat specialists among them. His remark doubtless made more of an impression on the former than the latter! And the man with lower-back pain who declined to see a gynaecologist for fear that he would advise a hysterectomy pointed up the lesson in spite of the absurdity of his humour.

We must seek the answers to such questions under conditions where many of the circumstances appear to be dependent on emotional factors. Men with superior education and training, will continue to be impatient with those who, by nature or nurture, have been denied this opportunity. The man who solves the puzzle will exult in his victory, even though others may have built up the case for him and made the outcome more certain. Too often the victor tries to enhance his position at the expense of those who tried and failed, whose limitations he disregards. Forgetfulness too can play its part as the consultant-specialist unloads the past from his mind. The dependence of the specialist on the general practitioner is less obligatory than the dependence of the general practitioner on the specialist.

The moral as well as the legal law governing malpractice is broad enough to harmonize the relationship, *if the virtues of group practice which we see at its best in the modern general hospital are placed in a dominant position. We must have no general practitioner whom we have not surrounded by a complete battery of specialists available to him, and we must have no specialist working independently of the general practitioner, except in those circumstances where he is justified in taking over in the best interests of the patient.* Thoughts like these can be incorporated in our code of ethics and in our organizational practices. In this respect *the hospital plays a key role in the organization of an extra-mural (Home Care) programme, in addition to its intra-mural programme on an integrated and continuing basis.* It can remove with one stroke the ugly consequences brought about by inequalities of opportunity and education which intensify and keep burning the class struggle in medical practice.*

In the hospital we see group practice at its best and *it is in the extra-mural direction of its activity that we have our optimum opportunity to expose the general practitioner to the highest grade of medical practice by sustaining the high level of his medical education.* Through such a plan, organizational differences and their consequences are minimized and each physician becomes more susceptible to the respect to which he is entitled. In this way we will at least have removed the predisposing, if not the exciting, causes for friction among physicians at the expense of the sick. The general and the special thus become interdependent, inseparable and, in fact, indivisible, each man contributing what he can contribute best. Setting the pace, the hospital can increase the wealth by sharing it, and the general practitioner who serves in the rank and file can be restored to his honourable place as the defender of the medical faith.

* See "The Principles and Practice of Home Care", *Journal of the American Medical Association*, 14th August, 1954, vol. 155, p.p. 1379–82.

